# The Evaluation of Egg-Parasitic Fungi *Paraboeremia taiwanensis* and *Samsoniella* sp. for the Biological Control of *Meloidogyne enterolobii* on Chinese Cabbage

**DOI:** 10.3390/microorganisms8060828

**Published:** 2020-05-30

**Authors:** Yu-Jie Liang, Hiran A. Ariyawansa, J. Ole Becker, Jiue-in Yang

**Affiliations:** 1 Department of Plant Pathology and Microbiology, National Taiwan University, Taipei 10617, Taiwan; b05613029@ntu.edu.tw (Y.-J.L.); ariyawansa44@ntu.edu.tw (H.A.A.); 2 Department of Nematology, University of California, Riverside, CA 92507, USA; obecker@ucr.edu

**Keywords:** *Meloidogyne enterolobii*, *Paraboeremia taiwanensis*, *Samsoniella* sp., biological control, nematophagous fungi, egg parasitism

## Abstract

*Meloidogyne enterolobii*, an aggressive plant-parasitic nematode, has been causing great yield loss worldwide in recent years. With no resistant Chinese cabbage cultivar available currently, a biological control strategy is needed to offer an eco-friendly option for sustainable farming. In this study, the nematode suppression efficacy of two newly isolated fungi, *Paraboeremia taiwanensis* and *Samsoniella* sp., were evaluated against *M. enterolobii* and compared to the known biological control agents *Hyalorbilia oviparasitica* strain DoUCR50 and *Purpureocillium lilacinum* strain 251 (PL251). Both *P. taiwanensis* and *Samsoniella* sp. reduced 29–63% disease severity as effectively as the commercial product PL251 on Chinese cabbage in greenhouse trails. The in vitro egg infection rate was 47.83% by *P. taiwanensis* and 47.50% for *Samsoniella* sp., respectively. A special protocol for scanning electron microscope observation of the fungi-infected nematodes was established in this study, and the egg parasitism of the four fungi against *M. enterolobii* was further confirmed. For all fungi examined in this study, fungal hyphae were seen apparently penetrating into *M. enterolobii* eggs without destructive damage of the overall outer eggshell and the hyphae continued to grow within eggs after 6 days of infection. The results of this study imply a similar egg-parasitism mechanism for *P. taiwanensis*, *Samsoniella* sp., *H. oviparasitica* DoUCR50, and *P. lilacinum* PL251. It further enlightens the application potential of nematophagous fungi as biocontrol agents against plant-parasitic nematodes in vegetable crop management.

## 1. Introduction

Root-knot nematodes (*Meloidogyne* spp.) have a significant impact on agriculture. They are distributed widely in tropical and subtropical regions and parasitize a broad host range, including about 3000 species of vegetables, ornamental plants, fruits, and weeds [1,2]. They are economically important plant-parasitic nematodes, which cause world-wide annual losses of approximately US$ 157 billion [3]. The foliar symptoms caused by root-knot nematode parasitism are similar to those caused by abiotic and other biotic diseases, such as yellowing and stunting, so the disease incidence and yield losses are often underestimated [4]. A fast-spreading emerging species of root-knot nematodes, *Meloidogyne enterolobii*, was included in the A2 alert list by the European and Mediterranean Plant Protection Organization (EPPO) in 2008 [5] and in the quarantine pest list of the Japan Plant Protection Station in 2020 [6]. Significant yield losses of guava in Thailand and losses of sweet potato in the US States of South and North Carolina by this nematode species were reported [7,8,9]. In Brazil, *Meloidogyne enterolobii* lead to a 70% yield reduction of guava within 7 years, causing an estimated direct loss of US$ 61 million [10]. Furthermore, varieties of important economic crops and rootstocks, including tomato, banana, cherry, grape, fig, and melons, were reported to be excellent hosts for the nematode [11]. Also, the disease was found to impact some organic farms planted with Chinese cabbage and guava in Taiwan, and the egg-masses are generally larger than the ones of *Meloidogyne incognita*. The potential impact of *M. enterolobii* in both tropical zones and areas with cooler temperature implies a worrisome global agriculture threat [7,12].

Control strategies for root-knot nematodes generally include chemical nematicide application, planting resistant cultivars, and some integrated management procedures that rely on cultural practice adjustments and the use of microbial enemies as biological control agents. However, to date, validated options for *M. enterolobii* management have been limited. Soil fumigants such as 1,3-dichloropropene and chloropicrin showed promising efficacy on reducing root galling and the nematode reproduction potential of *M. enterolobii* [13] but impose a potentially high risk to the environment and human health [14]. Resistant tomato cultivars with the *Mi* gene show resistance to *M. incognita*, *M. javanica*, and *M. arenaria*, but are susceptible to *M. enterolobii* [15]. With limited information on *M. enterolobii*’s host range, effective crop rotation plans are currently restricted to examined crops. Therefore, the development of biological control methods for *M. enterolobii* has become a reasonable choice. Various nematophagous fungi that feed on and destroy nematodes have been considered useful biological control agents [16]. They can be characterized into four groups based on their mode of action: (1) nematode-trapping; (2) parasites of eggs, sedentary juvenile stages and females of endoparasitic nematodes; (3) endoparasitic; (4) toxin-producing [17]. To date, egg-parasitic fungal species *Purpureocillium lilacinum*, *Pochonia chlamydosporia*, and *Trichoderma harzianum* were shown to be effective against *M. enterolobii* [7,18].

Some entomopathogenic fungi, species that parasitize insects, were also reported to exhibit nematode-infection abilities. For example, *Metarhizium guizhouense* was found to inhibit egg hatching and cause high juvenile mortality on *M. incognita* [19]. In addition, strains of *Beauveria bassiana* are capable of suppressing root galling of *M. hapla* in greenhouse experiments [20]. Furthermore, in a recent study, *Paraboeremia taiwanensis* developing on a fruiting body of *Cordyceps ninchukispora* as well as *Polycephalomyces elaphomyceticola* showed the ability of parasitizing the eggs of *Meloidogyne graminicola*, in in vitro conditions [21]. Most entomopathogenic fungi infect their host mechanically and enzymatically. Fungal hyphae and appressoria were first produced to penetrate the host, and extracellular enzymes, like chitinase and protease, were then secreted to degrade insect cuticles [22,23,24]. Díaz-Godínez et al. [25] proposed that chitinase is secreted to assist hyphae penetration, and protease and lipase are induced afterward depending on the insect components. A similar egg-parasitism mechanism is shared between entomopathogenic fungi and nematophagous fungi. Nematode eggs consist of three layers, an inner lipid, a middle chitin, and an outer vitelline layer [26]. Some nematophagous fungi form appressoria on the host’s surface to penetrate females and eggs [26]. Various enzymes secreted by nematophagous fungi are also considered critical for degradations of nematode body structure [27].

To develop an effective and eco-friendly *M. enterolobii* management method for sustainable farming, the nematode-suppression ability of two newly identified fungi, *P. taiwanensis* and *Samsoniella* sp., which stood out from 26 fungal isolates in our previous screening against *M. enterolobii*, were investigated in the study. The specific aims of this study were to (1) evaluate the effectiveness of the two fungi on egg infection of *M. enterolobii*; (2) explore the infection mechanisms of the examined fungi; (3) determine the biological control application potential of those fungi to control *M. enterolobii* on a popular vegetable crop, Chinese cabbage, in Taiwan. The evaluation was further compared with *Hyalorbilia oviparasitica* DoUCR50 (syn. *Dactylella oviparasitica* DoUCR50) [28] and *Purpureocillium lilacinum* 251 (syn. *Paecilomyces lilacinus* strain 251) [29]. The former is a well-studied hyperparasitic fungus of eggs, sedentary juvenile stages, and females of sugar beet cyst nematodes, originally isolated from a naturally *Heterodera schachtii*-suppressive soil [30,31]. The latter fungal strain was commercialized in the US as MeloCon WG [29]. It was reported as being effective in controlling several plant-parasitic nematodes, including *M. enterolobii* [18].

## 2. Materials and Methods 

### 2.1. Nematode Culture

The root-knot nematode *Meloidogyne enterolobii* was isolated from a guava field in Taiwan and identified morphologically and molecularly with multiplex PCR following the protocol by Hu at al. [32]. Nematodes were propagated on mung bean (*Vigna radiate*) in a 28 °C growth chamber. Egg-masses were hand-picked by forceps and sterilized with 0.5% NaOCl for 1 min, and then washed three times with sterile water [21]. Afterward, eggs were incubated in autoclaved ddH_2_O in a 28 °C growth chamber for about 7 days. The 2^nd^ stage juveniles hatched from the eggs and were collected for later use.

### 2.2. Fungal Culture

Four fungi species were used in this study: *Paraboeremia taiwanensis* (NTUCC 17–013), *Samsoniella* sp. (NTUCC 18–159), *Hyalorbilia oviparasitica* strain DoUCR50, and *Purpureocillium lilacinum* strain 251. The *P. taiwanensis* strain is a recently identified new species [21] and was maintained on potato dextrose agar (PDA) at 28°C. Similarly, the *Samsoniella* sp. strain NTUCC 18–159 was isolated, identified in Taiwan in 2018, and cultured on PDA at 24 °C. Conidia of both fungi were collected from 7 to 14 days-old cultures in 0.1% Tween 20 and filtrated through two layers of cheesecloth to prepare for the conidia suspension. *H. oviparasitica* DoUCR50 was cultured on PDA at 24 °C for 21 days. The mycelium was scratched off and vortexed with beads in 0.1% Tween 20 to break the hyphae into small propagules to prepare inoculum, and colony-forming units (CFU) per milliliter were counted. *Purpureocillium lilacinum* strain 251 (PL251) (BioAct™ DC, Bayer Taiwan Company Ltd., Taipei, Taiwan) was a commercial product kept at 10 °C before using. On the day of the experiment, a suspension of *P. lilacinum* was made with tap water to achieve the concentration of 10^6^ conidia/mL following the manufacturer’s suggestion.

### 2.3. In Vitro Infection Experiments

50 surface-sterilized *M. enterolobii* eggs were placed on 1% water agar plates containing 1% ampicillin and on which the test fungi had been cultured for 5–10 days at 28 °C. After 7 days of incubation at the same temperature, the eggs were observed with a dissecting microscope. Eggs colonized by hyphae were considered infected. The hatching rate and the infection rate were recorded. Plates without fungi growing were used as control. Each treatment was carried out 3 times with 4 replicates each.

### 2.4. Scanning Electron Microscopy (SEM)

The fungal-infected *M. enterolobii* eggs were observed with SEM to further determine the nematophagous mechanism. The eggs were first prepared as described previously for the in vitro experiment. The eggs infected with fungi were washed from the culture plate and fixed overnight in 2.5% glutaraldehyde in 0.1 mol L^−1^ phosphate buffer (pH 7.2) at 4 °C. The specimens were rinsed 3 times in 0.1% phosphate buffer (pH 7.2) for 10 min each. The samples were post-fixed in 1% osmic acid for 2 h, then rinsed twice in 0.1% phosphate buffer (pH 7.2) for 10 min. The post-fixed specimens were dehydrated in a series of ethanol (30%, 50%, 70%, 80%, 90%, 95%, and twice in 100%) for ten minutes each. After the dehydrated specimens were further dehydrated in a critical point dryer (Z-3100, Polaron Ltd., East Sussex, England) with liquid CO_2_, the samples were coated with gold-palladium using an ion sputter coater (Sputter coating system, SPI Inc., West Chester, PA, USA) and observed with JSM-5600 (JEOL Ltd., Tokyo, Japan) (SEM) at 15–20 kV.

### 2.5. Greenhouse Trials 

Seeds of Chinese cabbage (*Brassica rapa*) were planted in 9 cm diameter pots containing sterilized growth medium (peat:perlite = 2:1) and maintained in a growth chamber with 16 h light per day and a constant temperature of 28 °C in a randomized complete block design. The conidia suspensions of *P. taiwanensis* and *Samsoniella* sp. strain NTUCC 18–159 were counted with a hemocytometer and each was adjusted to 10^6^ conidia/mL with sterile water. When the seedlings were 7 days old, four holes were created around each plant, and 1 mL conidia suspension was poured evenly into the holes. One week after fungi inoculation, 200 J2 were introduced into each pot. Soil temperature was monitored with HOBO (MX2200, Onset Ltd., Bourne, MA, USA), and the temperature accumulation data were used for nematode generation time estimation. Approximately one week before *M. enterolobii* produced eggs of the first life cycle (21 days after J2 inoculation), another milliliter conidia suspension was inoculated to enhance the effect. Plants were harvested when approximately 2 nematode generations were completed. Additionally, three sets of plants were included as controls: (1) fungi-only inoculation, (2) nematode-only inoculation, and (3) without fungi or nematode inoculation. There were 5 replicates per treatment in the trial. When the trial was repeated, the experiment was adjusted to provide a better evaluation for field-application potential. The examination scale was enlarged to 12-cm diameter pots, with 4 mL 10^6^ conidia/mL per pot fungal inoculum and nematode inoculum 1000 J2 per pot, and 8 replicates per treatment. In addition, *H. oviparasitica* DoUCR50 and *P. lilacinum* PL251 were included for comparison. The cabbage seeds in the second trial were obtained from a different batch because a fungal infection occurred in all of the treatments in the first trial and lead to a harvest 10 days earlier than the scheduled date. At harvest, cabbage roots were separated from the aboveground part and carefully washed with tap water. The root length, shoot height, and dry weight was measured. The number of galls and egg-masses were counted under a dissecting microscope, and the galling index was determined to represent the disease severity of *M. enterolobii* on cabbage. The scale of 1–9 was used to determinate the galling index by estimating the proportions of galled roots: 1 = no galls, 2 = 1–3%, 3 = 4–12%, 4 = 13–25%, 5 = 26–38%, 6 = 39–50%, 7 = 51–65%, 8 = 66–80%, 9 = 81–100% galls observed over the entire root system [33].

### 2.6. Data Analysis

For the in vitro infection experiments, all data of the egg hatching rate and the infection rate was analyzed through analysis of variance (ANOVA). Means were analyzed by the Fisher’s least significant difference (Fisher’s LSD) at *P* = 0.05 using the RStudio Version 1.1.463. For the greenhouse trials, the root gall number and egg-mass number were log_10_(x + 1) transformed, and all data were analyzed through analysis of variance (ANOVA). Means were separated by the Fisher’s least significant difference (Fisher’s LSD) at *P* = 0.05 using the RStudio Version 1.1.463.

## 3. Results

### 3.1. In Vitro Experiments

**Figure 1:**
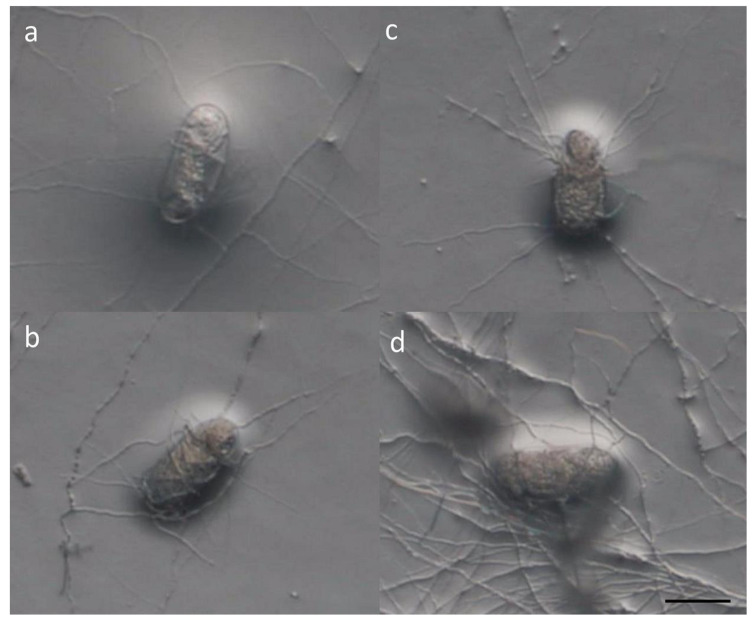
Observation of *M. enterolobii* eggs infected by nematophagous fungi, through a dissecting microscope. Bar = 45 μm. (**a**) *Paraboeremia taiwanensis,* (**b**) *Samsoniella* sp. strain NTUCC 18–159, (**c**) *Hyalorbilia oviparasitica* DoUCR50, (**d**) *Purpureocillium lilacinum* PL251.

The fungal infection rate and egg hatching of *M. enterolobii* in different fungal cultures were recorded. Hyphal-wrapped eggs could easily be observed under a dissecting microscope and could be distinguished from the unimpacted ones (Figure 1). The eggs with disintegration contents and obviously penetrated and parasitized by fungal hyphae were considered infected by the inoculated fungi. The infection rate represents the percentage of eggs that were parasitized by the examined fungus. All studied fungi were equally capable of infecting about 50% of eggs (Table 1). Quickly after fungal parasitism, the eggs were dead and could no longer hatch. Compared to the no-fungal control, all examined fungi reduced the hatching rate significantly (Table 1). The lowest egg hatching rate was seen in the *P. lilacinum* PL251 treatment (40.33%), which was less than half of what was observed in the control (86.67%). A 41.83% hatching rate was observed in the *H. oviparasitica* DoUCR50 treatment. As for *P. taiwanensis* and *Samsoniella* sp. strain NTUCC 18–159, the egg hatching rate was 46.33% and 47.16%, respectively. The egg hatching rate reflects the growth potential of the nematode population after treatment and is highly associated with the future damage on the host. The results indicated that all examined fungi are capable of parasitizing *M. enterolobii* eggs through penetration, and the survival of the nematode eggs were seriously impacted by these biological processes, which then led to significantly lower egg hatching rates.

### 3.2. Scanning Electronic Microscope (SEM)

**Figure 2:**
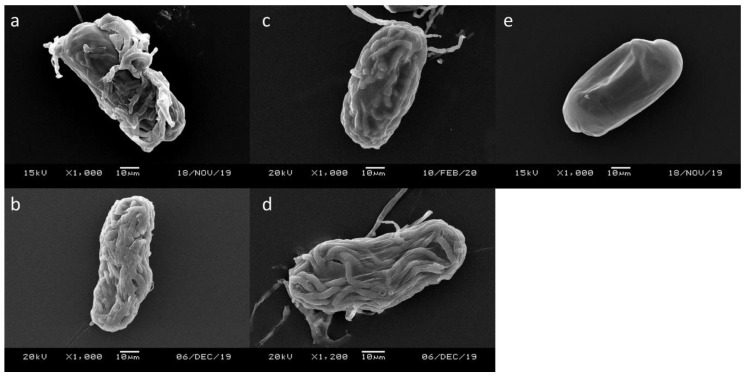
Observation of *M. enterolobii* eggs infected by nematophagous fungi through scanning electronic microscope (SEM). (**a**) *Paraboeremia taiwanensis*, (**b**) *Samsoniella* sp. strain NTUCC 18–159, (**c**) *Hyalorbilia oviparasitica* DoUCR50, (**d**) *Purpureocillium lilacinum* PL251, (**e**) No-fungi control.

Conventional SEM was used to observe the eggs infected by different fungi. The surface of the control eggs was smooth, and the shape of the first stage juvenile (J1) was visible (Figure 2e). For the eggs infected by the examined nematophagous fungi, the hyphae originally wrapped around the eggshell may be washed away during the preparation process. Still, the eggshells filled with fungal hyphae were clearly observed (Figure 2a–d). The fungal hyphae grew into the eggs and colonized them without totally disrupting the eggshell. The original egg contents were seemingly lost, but the eggs remained in shape; some hyphae grew out of the eggs. Based on the observation under SEM, the four fungi likely shared the same egg-parasitism mechanism.

### 3.3. Greenhouse Trials

**Figure 3:**
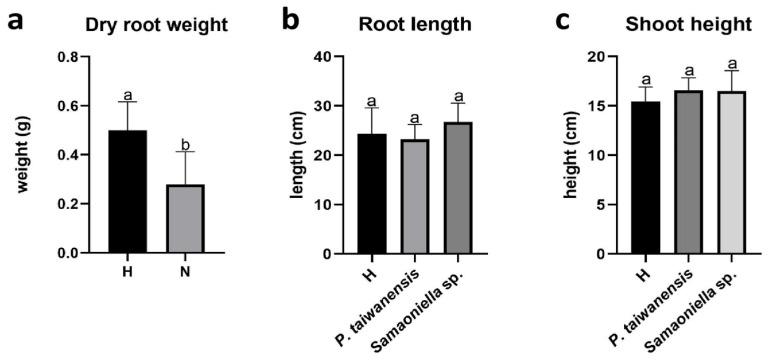
Plant growth parameters in greenhouse trial 1. (**a**) negative effect of *M. enterolobii* on dry root weight, (**b**) *P. taiwanensis* and *Samsoniella* sp. strain NTUCC 18–159 effects on root length, and (**c**) shoot height. H is the healthy control. N is the nematode-only treatment. Different letters indicate significant differences among treatments by Fisher’s LSD test (*P* < 0.05), respectively.

Dry root weights of the plant were significantly reduced when inoculated with nematodes (Figure 3a). No differences among all the treatments were observed in aboveground dry weight and shoot height. The examined fungi *P. taiwanensis* and *Samsoniella* sp. strain NTUCC 18–159 did not negatively affect the root length or shoot height when inoculated without the nematodes (Figure 3b,c). 

**Figure 4:**
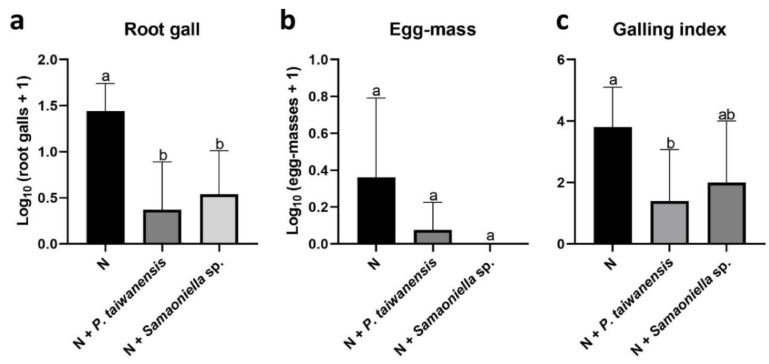
Effects of *P. taiwanensis*, *Samsoniella* sp. strain NTUCC 18–159, *H. oviparasitica,* and *P. lilacinum* strain 251 (PL251) on *M. enterolobii* in greenhouse trial 1. (**a**) root gall, (**b**) egg mass, (**c**) root galling index. N is the nematode-only treatment. Different letters indicate significant differences among treatments by Fisher’s LSD test (*P* < 0.05), respectively.

**Figure 5:**
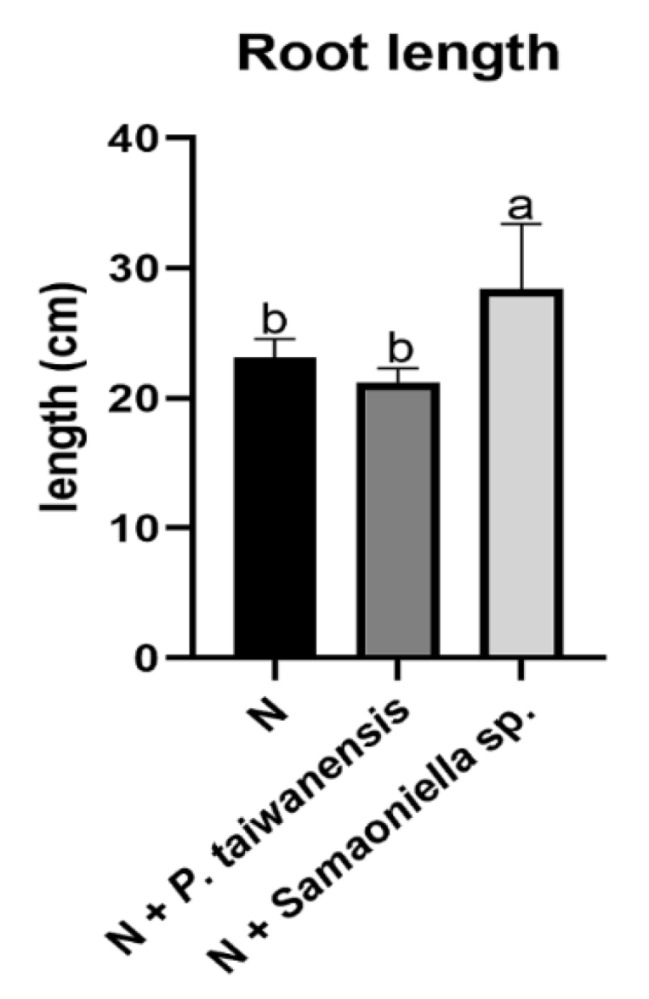
Root length promotion effect in greenhouse trial 1. N is the nematode-only treatment. Different letters indicate significant differences among treatments by Fisher’s LSD test (*P* < 0.05), respectively.

However, both *P. taiwanensis* and *Samsoniella* sp. strain NTUCC 18–159 inhibited the nematode reproduction. In the first trial, significant reductions (*P* < 0.05) were observed in root gall numbers in *P. taiwanensis* and *Samsoniella* sp. strain NTUCC 18–159 treatments (Figure 4a). Due to the 10-day earlier harvesting caused by a fungal infection, only a few egg-masses were observed. No significant reduction of egg mass numbers in fungal treatments was recorded (Figure 4b). Root galling index was reduced in the *P. taiwanensis* treatment but not with *Samsoniella* sp. strain NTUCC 18–159 (Figure 4c). Surprisingly, a root growth-promoting effect was observed on the plants co-inoculated with *Samsoniella* sp. strain NTUCC 18–159 and *M. enterolobii* (Figure 5).

**Figure 6:**
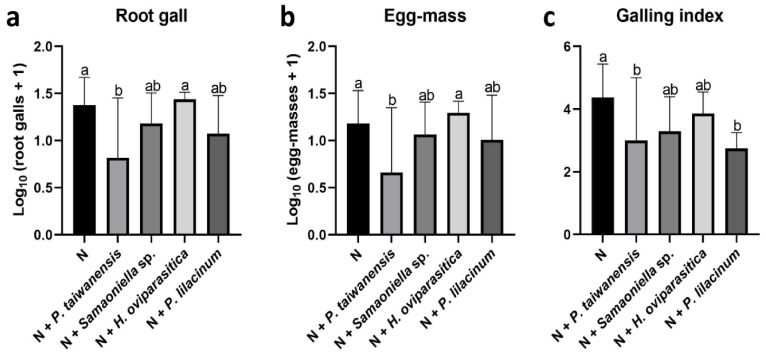
Effects of *P. taiwanensis*, *Samsoniella* sp. strain NTUCC 18–159, *H. oviparasitica* DoUCR50, and *P. lilacinum* strain 251 (PL251) on *M. enterolobii* in the second greenhouse trial. (**a**) root gall, (**b**) egg mass, (**c**) galling index. N is the nematode-only treatment. Different letters indicate significant differences among treatments by Fisher’s LSD test (*P* < 0.05), respectively.

**Figure 7:**
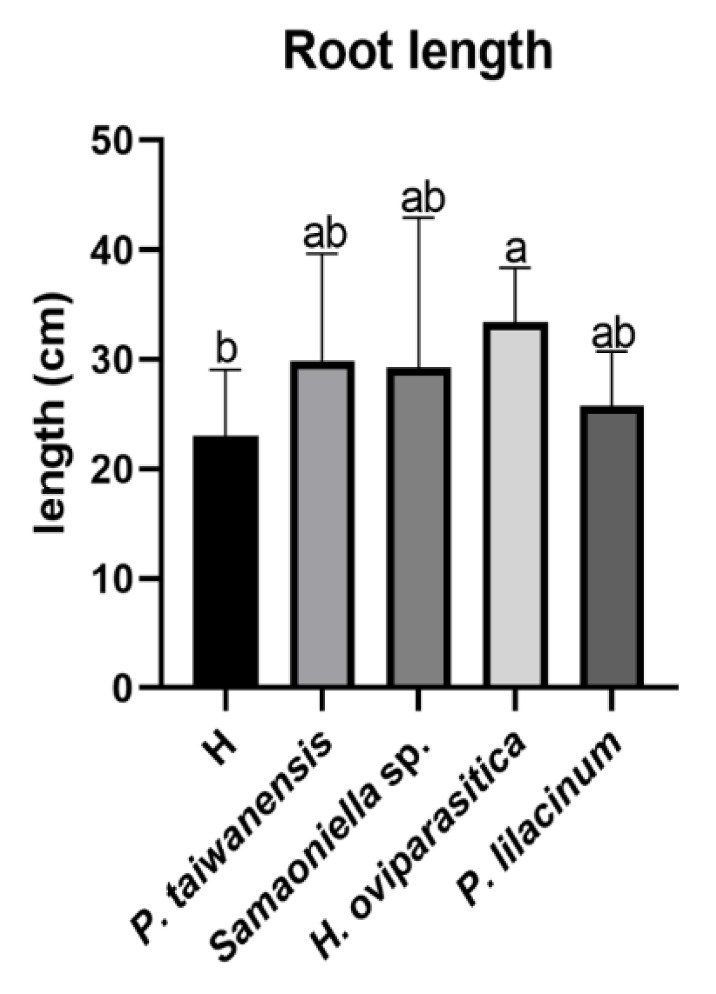
Root length promotion effect in the second greenhouse trial. H is the healthy control. Different letters indicate significant differences among treatments by Fisher’s LSD test (*P* < 0.05), respectively.

In the second trial, the *M. enterolobii*-suppression efficacy by *P. taiwanensis* and *Samsoniella* sp. strain NTUCC 18–159 were further compared with *H. oviparasitica* DoUCR50 and *P. lilacinum* PL251. Overall, the nematode-inhibition abilities of *P. taiwanensis* and *Samsoniella* sp. strain 180609-3 were similar to *P. lilacinum* PL251. The number of root galls significantly decreased in the *P. taiwanensis* treatment (Figure 6a). The reductions of root gall numbers were also observed in the two treatments inoculated with *Samsoniella* sp. strain NTUCC 18–159 and *P. lilacinum*, but were not significant by statistical standards (Figure 6a). The number of egg masses also showed a similar pattern (Figure 6b). A similarly galling index-reduction result was observed as in the first trial, the *P. lilacinum* treatment reduction was comparable to the *P. taiwanensis* treatment (Figure 6c). For the root and aboveground dry weights, there were no significant differences when compared to the no fungi-control. Similar results were seen in shoot height. For root length, no significant differences between healthy plants and plants infected by nematodes were observed, but *H. oviparasitica* alone increased the root length significantly (Figure 7).

## 4. Discussion

The *M. enterolobii*-suppression mechanism and ability of two newly identified fungi, *P. taiwanensis* and *Samsoniella* sp. strain NTUCC 18–159, were investigated through in vitro and in vivo experiments in the study. The overall results indicated the suppression efficacy was similar to *P. lilacinum* PL251. This particular strain had been widely commercialized and applied in fruit and vegetable cropping systems against root-knot nematodes, root-lesion nematodes, and citrus nematodes. Although there were some methodological differences, the egg hatching and infection rate in our study were similar to the previous in vitro research using *P. lilacinum* for controlling *M. enterolobii* [18]. As our data demonstrate, 40–50% *M. enterolobii* egg hatching rates with *P. lilacinum* PL251 in the in vitro experiments showed no significant differences with *P. taiwanensis* and *Samsoniella* sp. strain NTUCC 18–159. As for *H. oviparasitica* DoUCR50, previous studies reported its ability to parasitize primarily female sugar beet cyst nematodes [30], and a phylogenetically closely related fungal clade with similar nematophagous activity was identified [34]. This is the first study to reveal its ability to parasitize *M. enterolobii* eggs. However, in past observations, *H. oviparasitica* DoUCR50 hyphae would not penetrate eggs containing a juvenile (data not shown). This is consistent with *Metarhizium guizhouense,* another nematophagous fungi known to parasitize eggs of *Meloidogyne incognita* [19]. These phenomena suggest that the examined fungi are not capable of directly suppressing the egg hatching process. On the contrary, the fact that most of the non-hatched eggs in fungal treatments were penetrated by fungal hyphae indicates the principal parasitism mechanism was hyphae penetration. Further experiments on fungal secondary metabolites would provide valuable information on whether fungal secreted inhibitors, such as toxic substrates, are responsible of the gap between egg infection rate and reduction of egg hatching rate.

The interaction between *M. enterolobii* and the egg parasitic fungi was investigated with both a dissecting microscope and a scanning electron microscope (SEM), and the parasitism mechanism of the four nematophagous fungi was confirmed. With the dissecting microscope, the surfaces of the infected eggs were clearly seen wrapped by fungal hyphae. Through SEM, the hyphae growth and colonization inside the eggs were easily observed. The eggshells remained mostly in shape, but the contents within were nearly completely lost. This implied that these fungi secreted enzymes only to facilitate hyphae penetration while maintaining the egg contents inside the eggshell for the fungi to utilize the nutrients for later growth. After absorbing the nutrients, the hyphae grew out of the eggs and searched for a new host again. Similar mechanisms had been previously studied with the type strain of *H. oviparasitica*. Appressoria formed when the hyphae contacted the egg surface for attachment and penetration, and chitinase was known to be secreted by *H. oviparasitica* [35]. Moreover, Stirling and Mankau found that the hyphae of *H. oviparasitica* inside the eggs were swollen to 3–4 µm in diam compared to its normal width between 2–2.5 µm [36]. It was consistent with our observation that the *H. oviparasitica* DoUCR50 hyphae inside the eggs were about 4–4.5 µm in diam, which was thicker than hyphae located far from the eggs (around 2.5 µm in diam). It is worth mentioning that *H. oviparasitica* DoUCR50 is a strain with a different parasitic ability from Stirling and Mankau’s strain, but a similar mechanism was found when infecting *Meloidogyne* spp. However, such phenomena were not observed in the other three fungi in our investigation. Interestingly, entomopathogenic fungi shared similar mechanisms with egg-parasitic fungi when infecting insects. The appressoria structure and the enzymes secreted by fungi are considered as the crucial virulence factors. *P. lilacinum* had also been known to be both nematophagous and entomopathogenic [37,38,39]. The results from our previous [21] and this study proved that *P. taiwanensis* and *Samsoniella* sp. strain NTUCC 18–159 can both parasitize insects and nematodes, and the infection mechanisms appeared similar. Our findings not only showed the possibility of the two fungi being developed into biological control agents but also suggested that some entomopathogenic fungi might have a potential as biological control agents of nematodes.

The efficacies of the four fungi were further evaluated on Chinese cabbage in the greenhouse. Although the change of cabbage seeds might have been the reason why fewer egg masses were formed, the root gall numbers were reduced significantly, nevertheless. It clearly demonstrated the effectiveness of *P. taiwanensis* and *Samsoniella* sp. strain NTUCC 18–159 on inhibiting the nematodes’ feeding site establishment. The abilities of the two examined fungi to inhibit the *M. enterolobii* feeding site establishment and reproduction were as efficient as the already commercialized *P. lilacinum* PL251, despite the fact that only about 50% of eggs were parasitized by these fungi in vitro, which indicates the application potential as biological control agents. The galling index showed that the disease severity obviously decreased in *P. taiwanensis* and *P. lilacinum* treatments. However, the effects of *H. oviparasitica* DoUCR50 were not reflected in any of the nematode reproduction parameters. The weight and height of the Chinese cabbage indicated the actual market value of the crop, which was not impacted by any of the examined fungi. In summary, these fungi have the potential to become part of the integrated control strategies to root-knot nematode damage.

Many factors would influence the growth and control efficacy of biocontrol agents in planting materials or soil and therefore lead to efficacy gaps between in vitro study and greenhouse trials. Studies have shown that both inoculum level and application time affect the efficacy significantly [40]. Kiewnick and Sikora [41] mentioned that *P. lilacinum* cannot persist long in soil and it is likely necessary to apply 10^6^ cfu/g soil multiple times to achieve the inhibition effect on *M. incognita.* The concentration may also be applicable on *M. enterolobii*. The interaction of the fungi with other microbes in soil, such as competition, may be a crucial issue in its persistence, population size and, eventually, the success of nematophagous fungi [42]. Our greenhouse trials used a sterilized growth medium, and only one fungus was inoculated in each treatment. These were rather uniform environments that excluded many biological factors such as microbial interaction effects. Increasing the temperature and soil pH may also affect the parasitic capability of nematophagous fungi [43]. The in vitro testing was conducted on a culture medium of pH close to eight at room temperature, around 28 °C. Meanwhile, the planting materials created for the greenhouse experiments were under the conditions of pH 5.5–6.5 and an average temperature of 23 °C. In addition, specifically for *H. oviparasitica* DoUCR50, the growth rate of the fungus may be an important factor. Based on our observation, *H. oviparasitica* needed more than one month to colonize half of the 9-cm Petri dish with PDA, which is much slower than *P. taiwanensis*, *Samsoniella* sp. strain NTUCC 18–159, and *P. lilacinum* PL251. Although the growth rate may be different in soil, possibly *H. oviparasitica* DoUCR50 needed less time to reach the egg-masses and infect the eggs before the J1 developed. Therefore, a higher amount of inoculum might make up for its slow-growing feature and thus enhance its efficacy in soil. In addition, egg hatching would also be influenced by factors such as humidity, root exudates, and host age, resulting in the differences between in vitro test and greenhouse trial [44,45]; yet no study has been done specifically on this emerging new species, *M. enterolobii*. Regarding the problems related to the use of nematophagous fungi mentioned above, a cocktail of several nematophagous species may be the solution. Previous studies have supported the feasibility of such a concept. For example, Yankova et al. combined *Paecilomyces lilacinus* strain 251 and *Trichoderma viride* strain T6 and discovered the control efficacy was greater than using either one alone [46]. Different combinations of strains of *Pochonia chlamydosporia* var. *catenulata* and *Purpureocillium lilacinum* were also tested in the study by Silva et al. [18].

In conclusion, the egg-parasitism mechanism of *P. taiwanensis* and *Samsoniella* sp. strain NTUCC 18–159 was revealed in this study. The fungi showed potential as biocontrol agents against *M. enterolobii* as part of an integrated pest management (IPM) approach. Both *P. taiwanensis* and *Samsoniella* sp. strain NTUCC 18–159 are indigenous isolates in Taiwan. Given that such strains are often more receptive than non-indigenous ones [47], the application potential of the two in Southeastern countries where climatic conditions are similar is worth noting. Furthermore, previous studies of fungi within the *H. oviparasitica* clade and *Pochonia clamydospora* have supported the hypothesis that suppression efficacy of nematophagous fungi varies among strains [34,48]. Strains of *Purpureocillium lilacinum* obtained from eggs have greater potential to suppress nematodes than the ones from soil [49]. The continuation of searching for more effective *P. taiwanensis* and *Samsoniella* sp. strains of high phylogenetic similarity with the help of molecular tools seems to be a reasonable strategy in the future. In the meantime, field micro-pot trials with inoculations of a cocktail of nematophagous fungi against *M. enterolobii* will be conducted to optimize the application options and evaluate the ultimate control efficacy under microbial competition impacts.

## Figures and Tables

**Table 1:** Effects of the nematophagous fungi on *M. enterolobii* egg hatching and infection rate.

Fungi	Egg Hatching Rate (%) ^1^	Egg Infection Rate (%) ^1^
*Paraboeremia taiwanensis* NTUCC 17–013	46.33 ± 8.10 ^a^	47.83 ± 9.93 ^a^
*Samsoniella* sp. NTUCC 18–159	47.16 ± 8.13 ^a^	47.50 ± 9.18 ^a^
*Hyalorbilia oviparasitica* strain DoUCR50	41.83 ± 5.69 ^a^	51.17 ± 7.15 ^a^
*Purpureocillium lilacinum* stain PL251	40.33 ± 3.01 ^a^	55.83 ± 1.26 ^a^
No fungi control	86.67 ± 1.04 ^b^	-

^1^ Values refer to the means of 12 replicates. Means followed by the same letter in the same column showed no significant differences according to analysis of variance (ANOVA) with Fisher’s least significant difference (Fisher’s LSD) test (*P* ≥ 0.05).
